# *Phasic* and *repetitive* self-touch differ in hemodynamic response in the prefrontal cortex–An fNIRS study

**DOI:** 10.3389/fnrgo.2023.1266439

**Published:** 2023-11-29

**Authors:** Sabrina von Au, Ingo Helmich, Simon Kieffer, Hedda Lausberg

**Affiliations:** ^1^Department of Neurology, Psychosomatic Medicine and Psychiatry, Institute of Health Promotion and Clinical Movement Science, German Sport University (GSU) Cologne, Cologne, Germany; ^2^Department of Motor Behavior in Sports, Institute of Health Promotion and Clinical Movement Science, German Sport University (GSU) Cologne, Cologne, Germany

**Keywords:** self-touch, prefrontal activity, left orbitofrontal cortex, left dorsolateral prefrontal cortex, functional near-infrared spectroscopy

## Abstract

**Introduction:**

Each individual touches the own body several 100 times a day. While some researchers propose a self-regulatory function of self-touch, others report that self-touching increases nervousness. This controversy appears to be caused by the fact that researchers did not define the kind of self-touch they examined and actually, referred to different types of self-touch. Thus, kinematically defining different types of self-touch, such as *phasic* (discrete), *repetitive*, and *irregular*, and exploring the neural correlates of the different types will provide insight into the neuropsychological function of self-touching behavior.

**Methods:**

To this aim, we assessed hemodynamic responses in prefrontal brain areas using functional near-infrared spectroscopy (fNIRS) and behavioral responses with NEUROGES®. Fifty-two participants were recorded during three specific kinematically types of self-touch (*phasic, irregular, repetitive*) that were to be performed on command. The recently developed toolbox Satori was used for the visualization of neuronal processes.

**Results:**

Behaviorally, the participants did not perform *irregular* self-touch reliably. Neurally, the comparison of *phasic, irregular* and *repetitive* self-touch revealed different activation patterns. *Repetitive* self-touch is associated with stronger hemodynamic responses in the left Orbitofrontal Cortex and the Dorsolateral Prefrontal Cortex than *phasic* self-touch.

**Discussion:**

These brain areas have been reported to be associated with self-regulatory processes. Furthermore, *irregular* self-touch appears to be primarily generated by implicit neural control. Thus, by distinguishing kinematically different types of self-touch, our findings shed light on the controverse discussion on the neuropsychological function of self-touch.

## 1 Introduction

Self-touching is ubiquitous in everyday life (Grunwald et al., 2014; Kreyenbrink et al., 2017; Lausberg, 2022). According to observational studies, self-touch occurs in emotional, aroused, and stressful situations (Lausberg, 2013; Grunwald et al., 2014; Heubach, 2016; Kreyenbrink et al., 2017; Densing et al., 2018; Reinecke et al., 2020, 2022; Furley, 2021; Neumann et al., 2022). Some researchers propose a self-regulatory function of self-touch (Freedman et al., 1972; Grunwald et al., 2014; Helmich et al., 2014; Densing et al., 2018). Others report that self-touching represents stress. This controversy appears to be caused by the fact that researchers did not define the kind of self-touch they examined and actually referred to different types of self-touch (Reinecke et al., 2020). This leads to the fact that self-touch and its neuropsychological correlates are still poorly understood. Thus, kinematically defining different types of self-touch, such as *phasic* (discrete), *repetitive*, and *irregular*, and exploring the neural correlates of the different types will provide insights into the neuropsychological function of self-touching behavior.

Self-touch is defined as the dynamic physical contact between two parts of the body, typically the hand acting on a part of the body (Lausberg, 2022). Self-touch varies from scratching, rubbing, and kneading to stroking. Kinematically, based on the movement trajectory, three types of self-touch can be observed in everyday life and therefore distinguished as follows: *phasic* self-touches are characterized by a phase structure. They contain a transport phase, in which the hand is transported to the location of touching, and a concept phase, with a one-way movement path in which the hand acts on the body, which is directly followed by a retraction phase in which the hand is moved back, for example, a single stroke. *Repetitive* self-touches, such as *phasic* touches, consist of a transport phase, a concept phase, and a retraction phase. However, in the concept phase, the same movement path is used repetitively without a rest, for example, scratching. Only when a movement has been performed several times in the same direction does the retraction phase follow. In contrast, *irregular* self-touches have no phase structure. They are characterized by short movement paths in various directions and practically no displacement of the hand. Since they have no concept phase, they are not based on any motor plan (Lausberg, 2019). *Repetitive* vs. *phasic* touch represents two distinct phenomenological entities. It is not the quantity of a touch that is important but the quality of the contact (Spencer et al., 2003; Schaal et al., 2004; van Mourik and Beek, 2004; Huys et al., 2008; Lausberg, 2013). The different self-touch types occur in different contexts in daily life (Heubach, 2016; Mueller et al., 2019; Neumann et al., 2022). *Repetitive* self-touch is associated with better psychological wellbeing, in contrast to *irregular* self-touch (Reinecke et al., 2020). *irregular* self-touch probably serves to shield from other negative stimuli via strong somatosensory stimulation. Furthermore, opposite effects are found for *phasic* vs. *irregular* self-touch (Lausberg, 2022). *phasic* self-touch is also associated with the regulation process during acute stress and thereby enhances the cognitive process (Freedman and Bucci, 1981; Grunwald et al., 2014; Heubach, 2016). The higher the time proportion of *phasic* self-touching, the lower the subjective stress experience (Heubach, 2016). All three types of touch are to be distinguished in terms of their emotional, cognitive, and physical functions. In this case, it is not the quantity of a touch that is important but the quality of the contact (Lausberg, 2013). The differential effects of *repetitive, irregular*, and *phasic* self-touch explain the controversy debated by current researchers and show the importance of a fine-grained analysis of self-touch.

To our knowledge, there has never been any attempt to investigate brain activation during the three specific types of self-touch. Previous studies investigated self-touch without kinematically defining and distinguishing different types of self-touch. The self-touch was described as more “*repetitive*-like” or more “*phasic*-like,” but no specific movement criteria were used to classify self-touch. Different methods such as functional magnetic resonance imaging (fMRI) or electroencephalogram (EEG) were used to measure brain activity. These previous studies revealed a deactivation in the prefrontal areas, such as the ventrolateral prefrontal cortex, the orbitofrontal cortex (OFC), the dorsomedial prefrontal cortex, and the amygdala, the right striatum, the superior temporal gyrus, and the posterior cingulate for instructed explicit self-touch (Grunwald et al., 2014; Kikuchi et al., 2018; Boehme et al., 2019). These results are attributed to the principle of reafference. The reafference principle attenuates the effects of explicit and therefore conscious self-stimulation through predictive mechanisms (Weiskrantz et al., 1971; Blakemore et al., 1998; Synofzik, 2008; Boehme et al., 2019).

Studies have shown an association between *repetitive* movements in general and an activation in the prefrontal cortex (PFC). Brain activation of the PFC is considered to reduce arousal (Kinsbourne, 2011). Moreover, *repetitive* movements can lead to flow and trance-like states, where persons merge action and awareness and experience a loss of the sense of space and time (Hove and Stelzer, 2018; Sudeck and Thiel, 2020). The brain activation in the PFC and the OFC reflects the merging of action and awareness during flow (Nagai et al., 2004; Kinsbourne, 2011). The potential of *repetitive* movements to reach an extraordinary mental state such as flow implies that *repetitive* self-touch can have a strong self-regulatory effect. Considering this effect, it is important to differentiate between specific types of touch.

Research on social touch may further help with understanding the potential self-regulatory effect of self-touch. Social touches include all tactile touches that are not self-performed, regardless of whether these touches are performed directly skin-to-skin or not (Olausson et al., 2016). The neuropsychological effects of pleasantly perceived social touch, skin-to-skin and brush-to-skin, have been widely studied, and their positive effects on the recipient's wellbeing have been documented (Field, 2019; Li et al., 2019; Portnova et al., 2020; Uvnäs-Moberg et al., 2020). Unmyelinated C-tactile (CT) afferents in hairy skin are associated with these effects. These unmyelinated CT afferents are optimally activated at a *repetitive* gentle stroking frequency (1–10 cm/s; Field, 2019; Uvnäs-Moberg et al., 2020). Repeated activation of CT afferents is associated with better health by reducing sympathetic nervous system activity, increasing parasympathetic nervous system activity, and reducing stress, pain, and anxiety via oxytocin (Heinrichs and Domes, 2008; Ishak et al., 2011; Quirin et al., 2011; Love, 2014; Pfeifer et al., 2016; Walker et al., 2017; Hurlemann and Grinevich, 2018; Field, 2019; Uvnäs-Moberg et al., 2020; Uvnäs-Moberg and Petersson, 2022). At the neural level, correlates of social touch are found with the cortical brain regions in the dorsolateral prefrontal cortex (dlPFC) and the OFC, which are also associated with oxytocin projections (Rolls et al., 2003; Croy et al., 2016; Morita et al., 2018; Boehme et al., 2019; Field, 2019; Chen et al., 2020; Uvnäs-Moberg et al., 2020). Motor aspects of touch (discriminative touch) significantly predict the activation in the sensorimotor cortex (Rolls et al., 2003; Case et al., 2016). The higher the intensity of discriminative touch, the greater the change in signal (Kashou and Giacherio, 2016). However, research on affective touch indicates that brain activity shows a stronger association with pleasantness than with intensity in the context of affective touch (Case et al., 2016). Since these effects take place via tactile stimulation by hand or brush, it can be assumed that self-touch also may achieve stimulation of the CT afferents.

Research on non-verbal behavior and recent advances in neuroimaging research have shown that it may be of particular interest to incorporate more naturalistic conditions (Dehais and Ayaz, 2019; Mueller et al., 2019; von Lühmann et al., 2021). In the present study, this was taken into account by considering the three specific kinematic types of self-touch, which can be observed in everyday life. Furthermore, functional near-infrared spectroscopy (fNIRS), as a wearable neuroimaging system, enables data from freely moving participants. This allows participants to behave in a more naturalistic way during the self-touch (von Lühmann et al., 2021). This study leads to a more complex and innovative experimental paradigm.

Taken together, the main purpose of this study is to provide profound insights into how the healthy brain works. The aim of this study is to better understand the neural correlates of self-touch as a non-verbal behavior and to be able to solve the controversy through research. Our main hypothesis postulates that the three specific types of self-touch differ in their cerebral activity in the dlPFC and the OFC. Furthermore, we expect to observe higher brain activation for *repetitive* and *phasic* compared to *irregular* self-touch in the OFC and the dlPFC as an effect of *repetitive* and *phasic* self-touch being more involved in positive self-regulation. Owing to the fact that *repetitive* self-touch seems to have the highest impact on wellbeing, we further expect to observe higher brain activation for *repetitive* self-touch compared to *phasic* self-touch in the OFC and the dlPFC.

## 2 Materials and methods

To achieve high standards, this publication followed best-practice recommendations for fNIRS articles (Yücel et al., 2021).

### 2.1 Ethical approval

The study was approved by the Local Ethics Committee of the German Sport University (Nr. 162/2022). Written informed consent was obtained from each participant.

### 2.2 Participants

Fifty-two healthy individuals (mean age: 26.73 ± 5.23 years; 36 women, 16 men; note that a diverse sample was not achieved) participated in the study. According to a power analysis with G^*^Power, data for 43 participants were needed for a moderate effect size, a power of 0.8, and a low correlation (*f* = 0.25; α error probability = 0.05; power = 0.8; *r* = 0.2). All participants had no known history of neurological or psychiatric disorders. Handedness was examined using the Montreal Handiness Questionnaire version that Crovitz and Zener (1962) used at the Montreal Neurological Institute. Thirty-one participants were right-handed, and 21 were ambidextrous.

### 2.3 Experimental procedure

In a room with lowered blinds, participants were seated in a comfortable upright position on a chair without armrests to allow unrestricted arm and hand movements. To achieve familiarization with the experimental procedure, the participants practiced *phasic, repetitive*, and *irregular* self-touch. First, the three types of self-touch were presented in a tutorial video. After watching the video tutorial, the participants were instructed to perform the three types of self-touch on the upper side of the forearm as naturally as possible and as pleasantly as possible. Since research about affective touch indicates that brain activity shows a stronger association with pleasantness than with the intensity in the context of affective touch, the study focuses on the participants' natural and pleasant touch behaviors (Case et al., 2016). The participants immediately received feedback from the experimenter if their performance was in line with the definition of the values from NEUROGES®.

**Figure 1:**
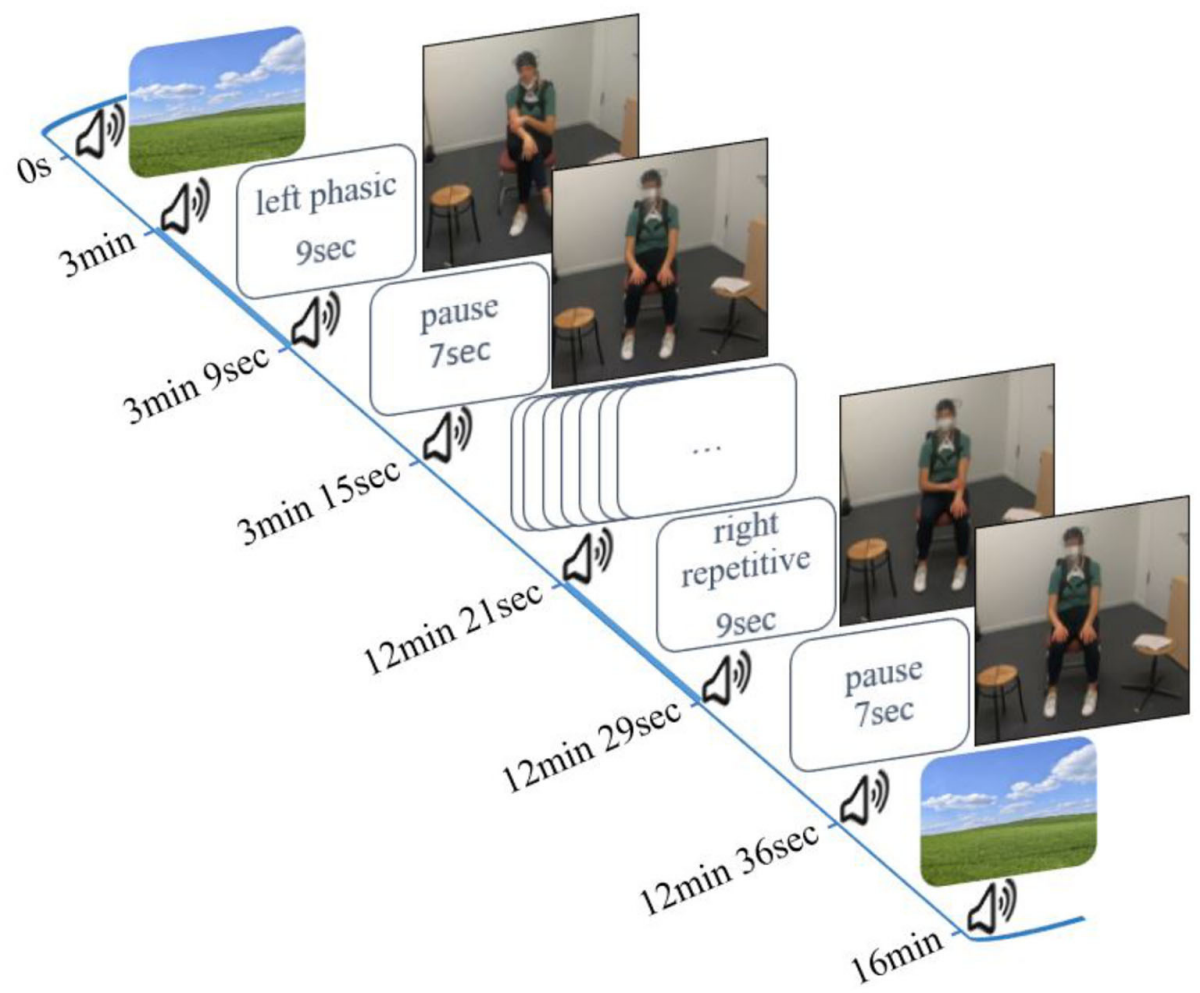
Study design and experimental setup.

To test our hypotheses, we used three conditions on command in a block design: (1) *phasic*: a single movement with no repetition of the same trajectory, (2) *repetitive*: same trajectory is executed at least twice; and (3) *irregular*: small *irregular* and unrhythmic movements (see the Introduction section). Each stimulus was performed six times with the right hand and six stimuli were performed with the left hand (left *phasic*–right *phasic*–left *irregular*–right *irregular*–left *repetitive*–right *repetitive*). This resulted in a total duration of 106 s per condition. The entire duration of the experiment was 16 min (Figure 1). *phasic* self-touch was performed first because this type of self-touch is expected to have the least sustained effects on cerebral activity (Lausberg, 2019). Since *phasic* self-touch and *irregular* self-touch hypothetically have the least cerebral similarity they followed sequentially (Schaal et al., 2004; Lausberg, 2013; Konczak and Winter, 2020). *Repetitive* self-touch seems to have the greatest and most lasting impact on cerebral activity in the regions of interest (ROIs). Therefore, *repetitive* self-touch was performed last. To ensure that the activation of the PFC can be attributed as little as possible to motor behavior, our design took into account two aspects. First, we presented familiar motor behavior stimuli (Lausberg, 2013; Heubach, 2016; Kreyenbrink et al., 2017; Densing et al., 2018; Reinecke et al., 2020; Neumann et al., 2022). Second, we presented each stimulus six times in a row. Thus, the stimuli did not come unexpectedly. Both aspects result in less involvement of the PFC (Miller and Cohen, 2001; Corbetta and Shulman, 2002; Lin et al., 2022).

The experiment started and ended with a 3-min resting phase in which the participant watched a neutral landscape presented on the screen. Participants began to move immediately after a peep sound, and the stimuli were given by written instructions (e.g., left *phasic*). They continued for 9 s until the next peep sounded and the word *pause* appeared on the screen. *Pause* means a resting phase without movement for 7 s (interstimulus interval). All instructions were developed with PsychoPy Version 3 (Peirce et al., 2019).

### 2.4 fNIRS acquisition and analyses

#### 2.4.1 fNIRS acquisition and montage

**Figure 2:**
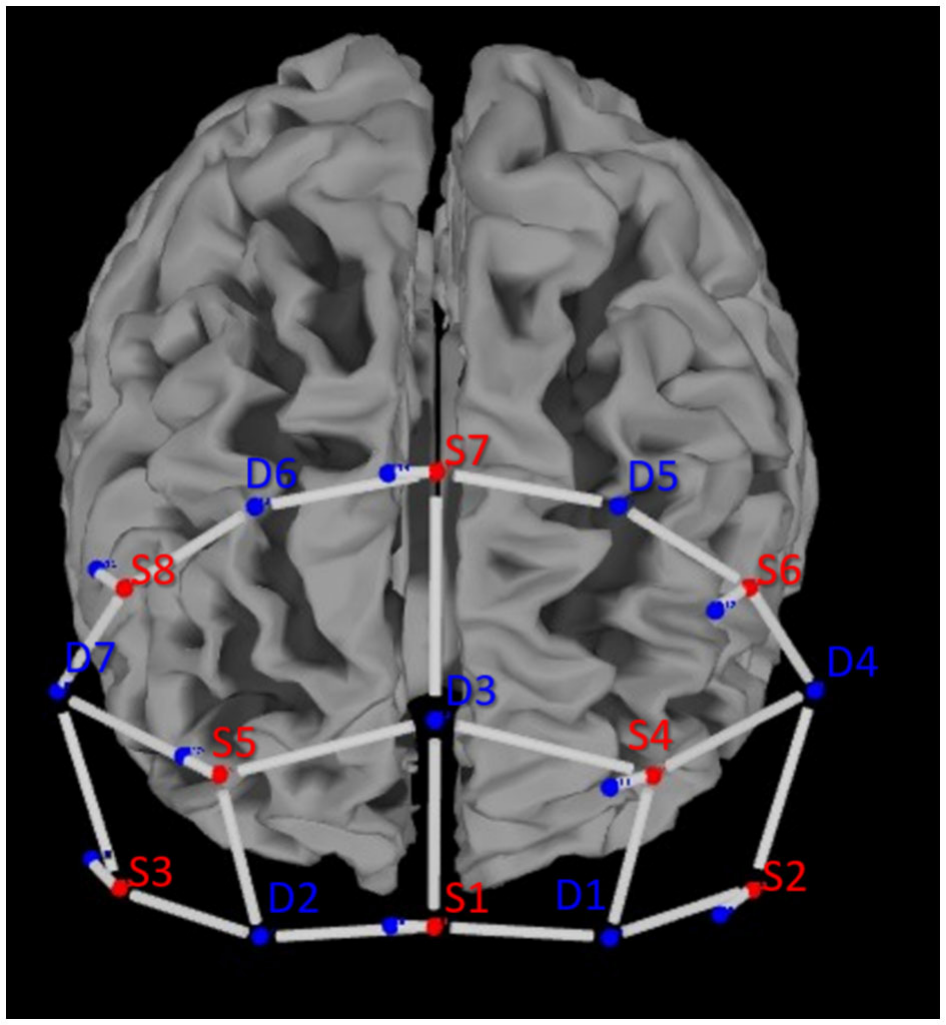
Optode placement according to the 10–20 system above the prefrontal cortex of the left and right hemispheres.

Cerebral activity was recorded using a portable continuous wave fNIRS system (NIRSport 2, NIRx, Medical Technologies LLC, Berlin, Germany; wavelengths of 760 and 850 nm; sampling rate 10.2 Hz). The montage contains eight light sources, seven detectors, and a bundle of eight short-distance detectors. The optodes were placed according to the 10–20 system on a standardized cap (EasyCap GmbH, Herrsching, Germany; Jasper, 1958). An fNIRS Optoden Location Decider was used to determine the most sensitive placement for each optode, transcribe the Montreal Neurological Institute and Hospital (MNI) coordinates, and determine the overlap of each channel with the corresponding brain region (>80% overlap; Morais et al., 2018; Table 1; Figure 2). The position of optodes allowed the coverage of brain areas in the prefrontal cortex. These areas included two ROIs in the frontal lobe: the OFC and the dlPFC (McGlone et al., 2012; Gordon et al., 2013; Bennett et al., 2014; Scheele et al., 2014; Morais et al., 2018; Table 1).

**Table 1:** fNIRS channels and corresponding brain regions.

**Channel**	**Optode names**	**MNI position**			**BA**	**Anatomical locations (% of overlap)**
		***x***	***y***	***z***		
CH1	S1–D1	−12	67	0	10 11	Left frontopolar area (55) Left orbitofrontal area (45)
CH2	S1–D2	13	67	0	10 11	Right frontopolar area (55) Right orbitofrontal area (45)
CH3	S1–D3	1	64	14	10	Frontopolar area (88)
CH5	S2–D1	−33	59	−2	11 46 10	Left orbitofrontal area (33) Left dorsolateral prefrontal cortex (25) Left frontopolar area (25)
CH6	S2–D4	−47	46	6	45 46	Left pars triangulari Broca's area (49) Left dorsolateral prefrontal cortex (43)
CH8	S3–D2	34	59	−2	10 11 46	Right frontopolar area (31) Right orbitofrontal area (31) Right dorsolateral prefrontal cortex (20)
CH9	S3–D7	48	46	5	45 46	Right pars triangularis Broca's area (44) Right dorsolateral prefrontal cortex (43)
CH11	S4–D1	−24	63	9	10 11	Left frontopolar area (70) Left orbitofrontal cortex (20)
CH12	S4–D3	−12	62	23	10 9	Left Frontopolar area (76) Left dorsolateral prefrontal cortex (15)
CH13	S4–D4	−39	50	17	46 45	Left dorsolateral prefrontal cortex (49) Left pars triangularis Broca's area (32)
CH15	S5–D2	25	63	9	10 11	Right frontopolar area (69) Right orbitofrontal cortex (22)
CH16	S5–D3	13	61	24	10 11	Right frontopolar area (73) Right dorsolateral prefrontal cortex (17)
CH17	S5–D7	40	50	16	46 45 10	Right dorsolateral prefrontal cortex (47) Right pars triangularis Broca's area (31) Right frontopolar area (19)
CH19	S6–D4	−46	39	26	45 46	Left pars triangularis Broca's area (73) Left dorsolateral prefrontal cortex (22)
CH20	S6–D5	−31	39	41	9 46	Left dorsolateral prefrontal cortex (67) Left dorsolateral prefrontal cortex (25)
CH22	S7–D3	2	50	39	9 10	Dorsolateral prefrontal cortex (62) Frontopolar area (20)
CH23	S7–D5	−9	41	50	9 8	Left dorsolateral prefrontal cortex (63) Left includes frontal eye fields (35)
CH24	S7–D6	10	41	50	9 8	Right dorsolateral prefrontal cortex (69) Right includes frontal eye fields (29)
CH26	S8–D6	30	40	41	9 46	Right dorsolateral prefrontal cortex (68) Right dorsolateral prefrontal cortex (22)
CH27	S8–D7	46	38	24	45 46	Right pars triangularis Broca's area (71) Right dorsolateral prefrontal cortex (24)

fNIRS, functional near-infrared spectroscopy; MNI, Montreal Neurological Institute and Hospital; BA, brodmann area.

Data were recorded from 20 long-distance channels of measurement and 8 short-distance channels to account for changes in the extracerebral blood flow (see Figure 2). The mean source–detector distance (long-distance channels) was 34.4 ± 4.6 mm. The short-distance channels were 8.0 ± 0.0 mm (Brigadoi and Cooper, 2015).

#### 2.4.2 Data quality check

To assess the quality of the fNIRS signal, data were visually inspected for each participant. To complete the visual inspection, the scalp coupling index (SCI) was computed during preprocessing (Pollonini et al., 2016). All channels with a value of <0.6 were rejected. Using this quality check, seven participants were excluded from further analysis. Of the remaining 52 participants, 46 had 0 rejected channels, 6 had 1 rejected channel, and 1 had 2 rejected channels.

#### 2.4.3 Preprocessing

The fNIRS data were analyzed using the Satori (v.1.8) toolbox (Lührs and Goebel, 2017). The preprocessing was performed on the whole signal for each participant. First, fNIRS raw data were transformed into optical density. Second, the SCI channel rejection was computed. Then, optical density was converted via the modified Beer–Lambert law (MBLL) into the concentration changes of oxygenated hemoglobin (Δoxy-Hb) and deoxygenated hemoglobin (Δdeoxy-Hb). Motion artifacts were corrected by applying the motion correction functions of Satori [spike removal; temporal derivative distribution repair (TDDR) according to Fishburn et al. (2019)]. Because the use of short-separation detector measurements as a regressor in the general linear model (GLM) has been previously shown to statistically improve hemodynamic response function (HRF) estimation (Gagnon et al., 2011; Yücel et al., 2015; Tachtsidis and Scholkmann, 2016), we used short-distance signals to regress out signals of extra-cerebral layers from the long-distance channels. To account for cardiac oscillations and Mayer waves, we used a 0.5 Hz low-pass filter, a high-pass filter (Butterworth) of 0.01 Hz, and the linear detrending function of Satori. The data were *z*-transformed.

#### 2.4.4 Statistical analyses

Since this study focused on naturalistic behavior, there was no pre-established number of repetitions or speed. Thus, we decided that the fNIRS response amplitude is better explained using a regressor based on duration compared to a regressor modulated by stimulus intensity.

The beta values were estimated for each channel and participant. The beta weights represent the strength of each regressor on the amplitude of the hemodynamic response (Plichta et al., 2007; Pinti et al., 2018). The beta values of the oxygenated hemodynamic and deoxygenated response were estimated by a general linear model and were statistically analyzed by the mixed-model analysis of variance (ANOVA) using R studio software (Allaire, 2012; R Core Team, 2022). The mixed ANOVA included the within-subjects factors CONDITION (*phasic* vs. *repetitive* vs. *irregular*) and CHANNEL (CH1:CH28). While the between-subjects factors GENDER (male vs. female) and HANDEDNESS (right-handed vs. ambidextrous) were not subjects of our research question, they were controlled for since previous studies had shown their effect on non-verbal behavior and cerebral blood flow (Skomroch et al., 2013; Helmich and Lausberg, 2014; Zhang et al., 2020; Helmich et al., 2022). The significance level was set to a *p*-value of < 0.05. We applied corrected significance according to Greenhouse and Geisser (1959). First, an analysis was performed which averaged over all channels and then for each single channel. Whenever the interaction between the factors reached significance, *post-hoc t*-tests were performed by applying Bonferroni correction.

### 2.5 Behavioral analysis (NEUROGES®)

The participants' behavioral responses, i.e., the execution of *phasic, repetitive*, and *irregular* self-touch on command, were assessed with the NEUROGES system for non-verbal behavior and gesture (Lausberg, 2019). The complete research coding manual has recently been accepted for open-access publication. The interrater agreement of two independent, certified raters as measured with the modified Cohen's kappa EasyDiag (Holle and Rein, 2015) was for *phasic* (kappa = 0.67), *repetitive* (kappa = 0.67), and *irregular* (kappa = 0.77) self-touch.

## 3 Results

### 3.1 fNIRS

#### 3.1.1 GENDER and HANDEDNESS

The analysis revealed a significant effect for the between-subjects factor GENDER, *F*_(1,49)_ = 5.18, *p* = 0.027 but no effects for the interactions of GENDER^*^ CONDITION, GENDER^*^ CHANNEL, and GENDER^*^CONDITION^*^CHANNEL. Furthermore, there was no significant effect for the between-subjects factor HANDEDNESS or of any interaction with CONDITION or CHANNEL. Since GENDER and HANDEDNESS had no effect on the within-subjects factors CONDITION and CHANNEL, which were the subjects of our research question, in the following, we only report the results for the latter factors.

#### 3.1.2 CONDITION

Averaged over all channels, there was a significant effect of CONDITION on the Δoxy-Hb (*p* = 0.031). The highest Δoxy-Hb was found for *irregular* self-touch [emmean = 0.072, *SE* = 0.034, 95% CI = (0.004– 0.14)], followed by *repetitive* [emmean = 0.066, *SE* = 0.034, 95% CI = (−0.002–0.14)] and *phasic* [emmean = −0.040, *SE* = 0.032, 95% CI = (−0.02–0.10)] self-touches. However, none of the *post-hoc* analyses' results showed significant effects (*phasic* compared to *irregular, p* = 0.07; *phasic* compared to *repetitive, p* = 0.12; and *irregular* compared to *repetitive, p* = 1.00).

There were no significant effects for beta weights on Δdeoxy-Hb.

#### 3.1.3 CHANNEL^*^CONDITION

**Figure 3:**
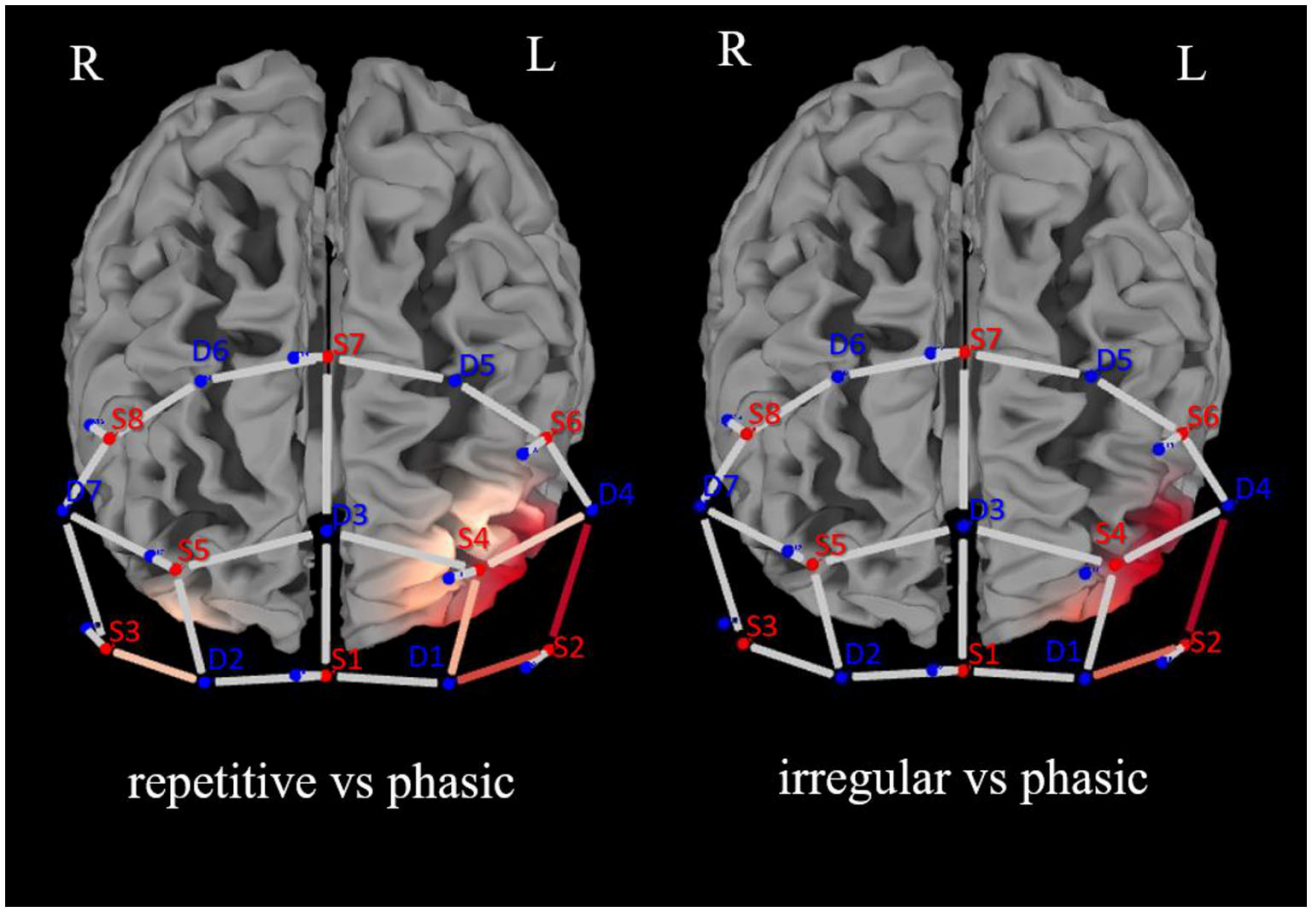
Brain activation (oxygenated hemoglobin) for the analysis of *repetitive* vs. *phasic* and *irregular* vs. *phasic* self-touch conditions for each single channel in the prefrontal cortex (red represents contrast: the darker the red, the greater the contrast described).

There was a significant effect of the interaction CONDITION^*^CHANNEL on the Δoxy-Hb, *F*_(54, 2, 646)_ = 1.33, *p* = 0.05. *Post-hoc* analyses resolved by CHANNEL indicated a significant increase of activation in four channels, namely, 5, 6, 11, and 13, in the left prefrontal cortex (see Figure 3).

**Figure 4:**
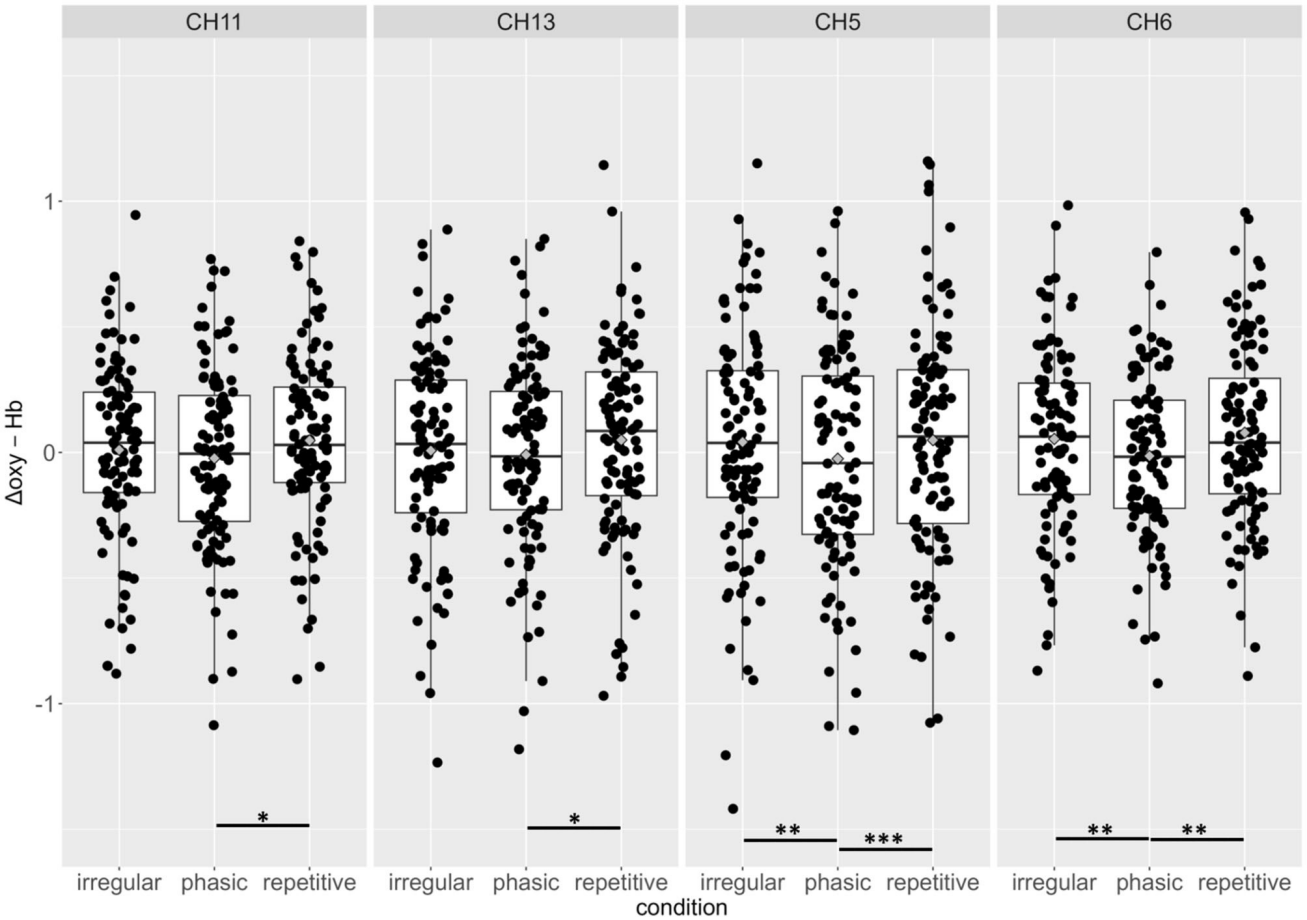
Group means and individual scores for oxygenated hemoglobin of the *irregular, phasic*, and *repetitive* conditions in channels 11, 13, 5, and 6. There were no significant effects for beta weights of Δdeoxy-Hb. *p < 0.05; **p < 0.01; ***p < 0.001.

In CH5 and CH6, significant differences between *phasic* and *repetitive* (CH5: *p* = 0.004; CH6: *p* = 0.001) and between *phasic* and irregular (CH5: *p* = < 0.001; CH6: *p* = 0.004) self-touch conditions were revealed, and in CH11 and CH13, there were also significant differences between *phasic* and *repetitive* (CH11: *p* = 0.037; CH13: *p* = 0.025) self-touch conditions. The contrast of *repetitive* vs. *irregular* self-touch conditions did not result in significantly different brain activation for oxygenated hemoglobin. The *post-hoc* analyses resolved by CONDITION showed no significant effects. The full details of analysis outputs are described in Table 2 and Figure 4.

**Table 2:** *Post-hoc* analysis of the CONDITIONS *repetitive* vs. *phasic* and *irregular* vs. *phasic* in the prefrontal cortex.

**Contrast**	**Channel**	***df***	***t*-ratio**	***p*-value**	**Anatomical regions**	**Hemisphere**	**BA**
*Phasic*–*repetitive*	13	49	−2.74	0.025	Dorsolateral prefrontal cortex Pars triangularis Broca's area	Left hemisphere	46
	11	49	−2.61	0.037	Frontopolar area Orbitofrontal cortex	Left hemisphere	10/11
	6	49	−3.81	0.001	Pars triangularis Broca's area Dorsolateral prefrontal cortex	Left hemisphere	45/46
	5	49	−3.39	0.004	Orbitofrontal area Dorsolateral prefrontal cortex Frontopolar area	Left hemisphere	11
*Phasic*–*irregular*	6	49	−3.38	0.004	Pars triangularis Broca's area Dorsolateral prefrontal cortex	Left hemisphere	45/46
	5	49	−4.10	0.0005	Orbitofrontal area Dorsolateral prefrontal cortex Frontopolar area	Left hemisphere	11

df, degrees of freedom; BA, brodmann area.

### 3.2 NEUROGES

The NEUROGES analysis of the participants' behavioral responses showed that *phasic* and *repetitive* self-touches were performed correctly, while only 108 of 1,872 *irregular* self-touches were performed correctly. The participants instead combined the specific types of self-touch. The participants performed *irregular* self-touches quite similarly to *repetitive* self-touches.

## 4 Discussion

The present study investigated the hemodynamic responses during the execution of different types of self-touch. The comparison of *phasic, irregular*, and *repetitive* self-touch conditions revealed different neural activation patterns, as measured by oxygenated hemoglobin, averaged over all channels. Furthermore, the analysis of the single channels revealed significant effects of *repetitive* self-touch within the frontopolar area, the orbitofrontal area, the dlPFC, and the pars triangularis Broca's area. In these brain regions, the execution of *repetitive* self-touch leads to stronger hemodynamic responses compared to *phasic* self-touch. The behavioral analysis, however, revealed that participants performed *phasic* and *repetitive* self-touch correctly but not *irregular* self-touch.

Despite practicing the different types of self-touch before the experiment, the participants, in the *irregular* condition, performed *irregular* self-touches quite similar to *repetitive* self-touches. This could be due to *irregular* self-touch being performed unconsciously, i.e., beyond the individual's awareness (Lausberg, 2022), in everyday life. The attempt to consciously perform this behavior, as requested in the present experiment, results in a more structured and regular performance. The more structured performance leads to self-touch that is more similar to that of *repetitive* self-touch. Therefore, in the present study, we cannot draw any conclusion from the fNIRS findings for the *irregular* condition about the neural correlates of *irregular* self-touch. The results of the *irregular* condition show that it is essential to distinguish between an implicit (naturalistic) self-touch paradigm and an explicit (on command) self-touch paradigm, which was performed in the present study (Grunwald et al., 2014). Furthermore, the results of the *irregular* condition show that the type of self-touch plays a crucial role in brain activity. Mostly, the factor time is considered with an increase in brain activation (Sailer et al., 2016). In terms of time, a difference between the *irregular* and the *phasic* condition would be expected. Therefore, the non-significant difference between these two conditions is due to the type of performance rather than due to the time factor. However, the following discussion concentrates on the fNIRS findings for the *phasic* and *repetitive* conditions.

The findings of the present study seem to be in contrast with those of previous studies about self-touch, which show a deactivation of or no effect on the brain during self-touch (Grunwald et al., 2014; Kikuchi et al., 2018; Boehme et al., 2019). Regarding our definition of the specific types of self-touch, Grunwald et al. (2014) investigated more “*phasic*-like” self-touch on command and Kikuchi et al. (2018) and Boehme et al. (2019) investigated more “*repetitive*-like” self-touch on command. Considering specific types of touch, our results are similar to Grunwald et al.'s (2014) but in contrast to those of Kikuchi et al.'s (2018) and Boehme et al.'s (2019) because the instructed *phasic* self-touch reveals no significant effects. However, for a clear understanding of previous research, a specific definition of self-touch and control of the participants' performance with objective and reliable behavioral methods would be necessary to enhance the reliability and interpretation of reported self-touch studies. Nevertheless, the present study is in line with the assumption that *repetitive* self-touch is able to have a stronger neuropsychological effect than *phasic* self-touch (Hove and Stelzer, 2018; Sudeck and Thiel, 2020).

Additionally, these results support the assumption that unmyelinated CT afferents are optimally activated with *repetitive* stroking (Field, 2019; Uvnäs-Moberg et al., 2020). Moreover, the execution of *repetitive* self-touch activated the left hemispheric pars triangularis Broca's area, the left hemispheric dlPFC, and the left hemispheric frontopolar and left hemispheric orbitofrontal areas. Concerning the field of social touch, the OFC and the dlPFC play important roles in self-regulation, and an association with oxytocin is likely (Heinrichs and Domes, 2008; Ishak et al., 2011; Kinsbourne, 2011; Love, 2014; Pfeifer et al., 2016; Walker et al., 2017; Hurlemann and Grinevich, 2018; Field, 2019; Portnova et al., 2020; Uvnäs-Moberg et al., 2020; Uvnäs-Moberg and Petersson, 2022). In particular, the activation of the left hemispheric dlPFC indicates an important structure for emotional processing and regulation (Herrington et al., 2005; Nejati et al., 2021). Hence, we reject the theory that self-touch via the reafference principle cannot have any effects (Weiskrantz et al., 1971; Blakemore et al., 1998). Instead, we underline the argument that self-touch is of behavioral relevance (Synofzik, 2008; Boehme and Olausson, 2022). We, therefore, assume that *repetitive* self-touch is similarly involved in self-regulation as social touch via the OFC and the dlPFC.

Given that our findings shed light on the controversial discussion about the neuropsychological function of self-touch, it is necessary to define specific types of self-touch conditions to achieve a deeper understanding of the neuropsychological correlates of human behavior and enhance the reliability and interpretation of reported self-touch studies. Thus, *repetitive* self-touch on command appears to indicate self-regulation processes, while *phasic* self-touch does not. Contrary to this finding, Grunwald et al. (2014) revealed that “*phasic*-self-touch” under an implicit paradigm is involved in self-regulation. Therefore, further studies should distinguish between an implicit and an explicit self-touch paradigm, and for reliable data, behavioral analysis methods should be used. Furthermore, additional physiological data such as heart rate variability or respiration measurements could help quantify the self-regulative processes more precisely.

Besides the above-discussed limitations, the sample size (*n* = 52) constitutes a particular strength of this study. In fact, most studies investigated smaller samples (Grunwald et al., 2014; Herold et al., 2018; Kikuchi et al., 2018; Boehme et al., 2019). Additionally, we revealed the first study that controls the participants' performance with objective and reliable behavioral methods. Thus, the large sample size and the in-depth study of self-touch combined with behavioral analyses are strengths of the present study. Furthermore, our innovative experimental paradigm includes more naturalistic stimuli. The more naturalistic stimuli provide information about the natural movement behavior.

### 4.1 Conclusion

To summarize, the present study conducted an innovative and complex experimental paradigm using fNIRS in combination with motor–behavioral analyses. For the first time, hemodynamic responses during specific non-verbal human behaviors were investigated in a large sample size. The experiment provided information on how the brain works in more realistic environments. We quantified neural correlates in the OFC and dlPFC during *repetitive* and *phasic* self-touch conditions. These brain regions, which have been described as being associated with self-regulatory processes, were activated to be significantly stronger during *repetitive* than during *phasic* self-touch. Thus, the present findings indicate that *repetitive* self-touch has a stronger self-regulatory function than *phasic* self-touch.

Methodologically, the present study further demonstrates that, when investigating the neural correlates of behaviors, it is important to control the participants' performance with objective and reliable behavioral methods. Regarding exploring the neural correlates of *irregular* movements, future studies should investigate *irregular* self-touch in an implicit paradigm such as real-world environments.

## Data availability statement

The original contributions presented in the study are included in the article/supplementary material, further inquiries can be directed to the corresponding author.

## Ethics statement

The studies involving humans were approved by Local Ethics Committee of the German Sport University (Nr. 162/2022). The studies were conducted in accordance with the local legislation and institutional requirements. The participants provided their written informed consent to participate in this study. Written informed consent was obtained from the individual(s) for the publication of any potentially identifiable images or data included in this article.

## Author contributions

SA: Conceptualization, Data curation, Formal analysis, Writing—original draft, Project administration. IH: Writing—review & editing, Formal analysis, Visualization. SK: Formal analysis, Writing—review & editing, Methodology. HL: Conceptualization, Supervision, Writing—review & editing.
